# Enhanced contextual fear memory in peroxiredoxin 6 knockout mice is associated with hyperactivation of MAPK signaling pathway

**DOI:** 10.1186/s13041-021-00754-1

**Published:** 2021-02-25

**Authors:** Sarayut Phasuk, Tanita Pairojana, Pavithra Suresh, Chee-Hing Yang, Sittiruk Roytrakul, Shun-Ping Huang, Chien-Chang Chen, Narawut Pakaprot, Supin Chompoopong, Sutisa Nudmamud-Thanoi, Ingrid Y. Liu

**Affiliations:** 1 Institute of Medical Sciences, Tzu Chi University, Hualien, Taiwan; 2 Department of Physiology, Faculty of Medicine Siriraj Hospital, Mahidol University, Bangkok, Thailand; 3 Department of Laboratory Medicine and Biotechnology, Tzu Chi University, Hualien, Taiwan; 4 National Center for Genetic Engineering and Biotechnology, National Science and Technology Development Agency, Pathum Thani, Thailand; 5 Institute of Biomedical Sciences, Academia Sinica, Taipei, Taiwan; 6 Department of Molecular Biology and Human Genetics, Tzu Chi University, Hualien, Taiwan; 7 Department of Anatomy, Faculty of Medicine Siriraj Hospital, Mahidol University, Bangkok, Thailand; 8 Department of Anatomy, Faculty of Medical Science, Naresuan University, Phitsanulok, Thailand; 9 Centre of Excellence in Medical Biotechnology, Faculty of Medical Science, Naresuan University, Phitsanulok, Thailand

**Keywords:** Peroxiredoxin 6, Trace fear conditioning, Fear memory, Posttraumatic stress disorder, MAPK signaling

## Abstract

Fear dysregulation is one of the symptoms found in post-traumatic stress disorder (PTSD) patients. The functional abnormality of the hippocampus is known to be implicated in the development of such pathology. Peroxiredoxin 6 (PRDX6) belongs to the peroxiredoxin family. This antioxidant enzyme is expressed throughout the brain, including the hippocampus. Recent evidence reveals that PRDX6 plays an important role in redox regulation and the modulation of several signaling molecules involved in fear regulation. Thus, we hypothesized that PRDX6 plays a role in the regulation of fear memory. We subjected a systemic *Prdx6* knockout (*Prdx6*^*−/−*^) mice to trace fear conditioning and observed enhanced fear response after training. Intraventricular injection of lentivirus-carried mouse *Prdx6* into the 3rd ventricle reduced the enhanced fear response in these knockout mice. Proteomic analysis followed by validation of western blot analysis revealed that several proteins in the MAPK pathway, such as NTRK2, AKT, and phospho-ERK1/2, cPLA2 were significantly upregulated in the hippocampus of *Prdx6*^*−/−*^ mice during the retrieval stage of contextual fear memory. The distribution of PRDX6 found in the astrocytes was also observed throughout the hippocampus. This study identifies PRDX6 as a participant in the regulation of fear response. It suggests that PRDX6 and related molecules may have important implications for understanding fear-dysregulation associated disorders like PTSD.

## Introduction

Fear acquisition and expression to threatening stimuli are innate responses to avoid dangers or predators to ensure safety and survival [1, 2]. Several pieces of evidence suggest that brain regions, including the amygdala, medial prefrontal cortex, and hippocampus, are required for an appropriate level of fear response [3–5]. Dysregulation of these brain regions leads to an excessive fear response in post-traumatic stress disorder (PTSD) [6]. The underlying molecular mechanism is still unclear. Peroxiredoxin 6 (PRDX6) is a multifunctional enzyme belonging to the peroxiredoxin superfamily [7]. Among the peroxiredoxin superfamily, PRDX6 is the only member that displays multiple functions, including the glutathione peroxidase (GPx), acidic calcium-independent phospholipase A2 (aiPLA2), and lysophosphatidylcholine acyltransferase (LPCAT) activities [7, 8]. These activities determine their roles in various organs under different physiological and pathobiological conditions [9, 10]. Although PRDX6 is expressed in various brain regions associated with fear regulation, including the hippocampus [11, 12] and expressed in all cell types with high expression level in the astrocytes [11, 13, 14], its function regarding cognition, particularly fear memory regulation has not yet been identified. Previous findings confirmed the association between enhanced fear memory and decreased overall enzymatic activity of GPx in the hippocampus, suggesting that GPx-PRDX6 may be involved in the regulation of fear response [15]. Besides, activation of PLA2 is required to acquire and retrieve emotional memory [16], indicating that aiPLA2-PRDX6 may also have a similar function. All the evidence mentioned above led us to hypothesize that PRDX6 may play an important role in regulating fear memory.

Trace fear conditioning (TFC) is a behavioral paradigm widely used to study associative fear memory [17]. The molecular mechanisms underlying fear memory processes are commonly approached with a fear conditioning paradigm, which shares similar mechanisms across species [18, 19]. This task causes fear memory formation by triggering a series of molecular and cellular changes to strengthen synaptic plasticity in emotion-related brain regions, including the hippocampus and the amygdala [20]. Tyrosine kinase receptor B (TrkB) and its downstream targets such as extracellular signal-regulated protein kinases 1 and 2 (ERK1/2) and protein kinase B (AKT) [21] are involved in the mediation of synaptic plasticity for fear memory formation. Interestingly, PRDX6 can modulate both ERK1/2 and AKT [22] expression, supporting our hypothesis that PRDX6 may participate in the neurobiological process of fear memory.

We performed behavioral, cellular, and molecular studies in the *Prdx6* knockout (*Prdx6*^*−/−*^) mice in the present study. We first identified the function of PRDX6 by employing *Prdx6*^*−/−*^ mice to trace fear conditioning (TFC) and found that this knockout strain exhibited enhanced contextual fear memory. We further confirmed with a gain-of-function study by injecting lentivirus-carrying mouse PRDX6 (mPRDX6) into the lateral ventricle of *Prdx6*^*−/−*^ mice, which mitigated their enhanced contextual fear memory. We also investigated their general behaviors using open field, three-chambers tests, marble burying, and elevated plus-maze. Proteomic and immunoblotting analyses were also performed in this study to understand the molecular mechanism better.

## Materials and methods

### Animals

All experiments on animals were approved by the Institutional Animal Care and Use Committee of Tzu Chi University, Taiwan (approval #104099), and complied with the Taiwan Ministry of Science and Technology guidelines for animals' ethical treatment. Twelve- to 14-week-old wild-type (C57BL/6J) and *Prdx6*^*−/−*^ mice were originally generated by Wang X. and colleagues and provided by Dr. Shun-Ping Huang at Tzu Chi University, Taiwan [23]. All mice were maintained in the Laboratory Animal Center of Tzu Chi University and were housed with ad libitum access to food and water under a constant 12-h light/dark cycle*.* Heterozygous knockout mice with one male and two females were crossed to reproduce *Prdx6*^*−/−*^ mice and their wild-type littermates. Genotyping (Additional file 1: Fig. S1a) was conducted to confirm the absence of the *Prdx6* gene in knock-out mice before every behavioral test. After the completion of trace fear conditioning, qRT-PCR (Additional file 1: Fig. S1b) and immunoblotting (Additional file 1: Fig. S1c) were carried out to visualize mRNA and protein level of PRDX6 in *Prdx6*^*−/−*^ mice. Moreover, we also recorded the morphology and bodyweight of the *Prdx6*^*−/−*^ mice. We found that both morphology (Additional file 1: Fig. S1d) and body weight (Additional file 1: Fig. S1e) (*t*_19_ = − 1.426, *p* = 0.170) of the *Prdx6*^*−/−*^ mice appeared to be normal.

### Behavioral tests

#### Trace fear conditioning (TFC)

Trace fear conditioning was modified from the protocol used in our previous study [17]. The conditioned chamber (17 cm (W) × 17 cm (L) × 25 cm (H)) illuminated with a white 30-lx light under the top-view camera was used in this study. After three days of habituation, mice were placed into the chamber for 2 min as a baseline and were then trained with three pairs of tone (CS) and electric foot shock (US) with an inter-trial interval of 1 min. One pair of CS-US consisted of a 20 s of tone (6000 Hz, 85 dB) followed by 1 s electric foot shock (2 mA) with a 10 s training interval. The mice were maintained in the conditioned chamber for a total of 9 min. To test their contextual fear memory retention, the mice were re-exposed to a conditioned chamber for 6 min without giving any tone and footshock after 24 h of the training session. One hour later, the mice were tested with cue fear memory by exposing them to 6 min of tone only after 1 min of habituation in an unconditioned context. The freezing behavior, defined as no movement except breathing, was analyzed using tracking software (EthoVision XT 15, Noldus Information Technology). The freezing time was converted to freezing percentage using the following formula:

$$\% {\text{Freezing}} = \left( {{\text{total freezing time}}/{\text{total test time}}} \right) \times 100.$$

#### Open field test

An open chamber (50 cm (W) × 50 cm (L) × 50 cm (H)) was used to test the locomotor function and anxiety-like behavior of the mice under the light-on condition [17]. The camera hung on top recorded the animals' locomotor activity within 10 min of the test. Their locomotor activity (distance traveled and moving speed) and time spent in the center and outer area were measured and analyzed by the tracking software (EthoVision XT 15, Noldus Information Technology).

#### Three-chambers test

This task was composed of three trials with 10 min of exploration time for each. The intertrial interval was 20 min. During the habituation trial, the experimental mice were placed into the middle compartment. Mice freely explored all three compartments that contained empty cups at the end of the left and right compartments. For the second trial, a sex- and age-matched stranger mouse (S1) was kept inside the cup in the right compartment. The experimental mice were then allowed to explore all compartments. For the third trial, another stranger mouse (S2) was placed in the cup located in the left compartment. The experimental mice were again placed in the middle compartment and allowed to explore the chamber. The time spent interacting with the empty cups or stranger mice was analyzed by tracking software (EthoVision XT 15, Noldus Information Technology). We followed the protocol described in a previous study [24].

#### Marble burying test

The protocol was described in a previous study [25]. Briefly, the cage (30 cm × 27 cm × 26 cm) was filled with 5 cm autoclaved bedding containing 20 marbles arranged centrally 4 by 5 and was kept in a soundproof box with 10 lx. Mice were placed and then filmed for 30 min. The number of unburied marbles was counted after 25 min.

#### Elevated-plus maze test

The elevated-plus maze is used to assess the anxiety-related behavior in rodents [26]. The apparatus consists of a "plus"-shaped maze at 60 cm height above ground with two oppositely positioned closed arms and two oppositely positioned open arms and a center region. The experiment was conducted during day time under the same light intensity (~ 130 lx) as provided in the animal housing room. The mice were placed in the center region facing one of the closed arms and allowed to explore the maze freely for 10 min. We used a video camera and tracking system (EthoVision XT 15, Noldus Information Technology) to record and analyze their anxiety-like behavior, respectively.

### Lentiviral vector preparation

Total RNA was isolated from the mouse hippocampus and converted to cDNA using oligo (dT) 18 primers. The cDNA was then amplified using a specific forward primer (5′-CTA GCTAGC ATG CCC GGA GGG TTG CTT C-3′ containing a NheI site) and reverse primer (5′-GC GAA TTC TTA AGG CTG GGG TGT ATA ACG-3′containing an EcoRI site) [52]. Full-length mouse *Prdx6* cDNA was purified by a PrestoTM Mini Plasmid Kit (catalog #PHD300, Geneaid Biotech Ltd., Taiwan). pLAS3wPpuro vectors containing *EGFP* and *Prdx6* were designed for the production of lentiviral vectors. HEK293T cell lines were used to produce lentiviruses containing either *EGFP* or *Prdx6* gene. Harvested lentivirus was concentrated using PEG-it (™) virus precipitation solution (System Biosciences, CA) and processed for titration.

### Stereotaxic surgery and intracerebroventricular injections of lentivirus containing mouse PRDX6

The procedures for stereotaxic injection were performed according to our previous study with slight modification [27]. The mice were anesthetized by intraperitoneal (IP) injection of ketamine/xylazine mixture (0.45 ml/25 g of body weight) and then fixed on the stereotaxic frame (Stoelting, US). The lentivirus containing either EGFP or mouse PRDX6 was dissolved in sterile 1× phosphate-buffered saline (PBS) to obtain the final titer of 7 × 10^5^ in 2 µl volume. The lentiviral vectors were then unilaterally injected into the right lateral third ventricle with the following brain coordinates: anterior–posterior (AP), − 0.5 mm; medial–lateral (ML), 1 mm (from the bregma): and DV, 2.33 mm (from the skull surface). A 10-µl Hamilton syringe with a 26 G needle was placed on the microinfusion pump (KD Scientific Inc. MA, USA) and connected via polyethylene—28 mm I.D. tubing to the internal cannula. We injected the lentiviruses with a flow rate of 0.5 µl/min over 4 min. The cannulas were placed for another 5 min to allow diffusion before removing them. Following surgery, mice were given pain killers (meloxicam, Achefree, Taiwan) and allowed to recover for 4 weeks before the behavioral tests.

### Liquid chromatography–tandem mass spectrometry (LC/MS–MS)

After completing a contextual test, protein samples were collected from the whole hippocampi of *Prdx6*^+*/*+^ and *Prdx6*^*−/−*^ mice. Protein samples from 3 mice were pooled together for each group and measured the protein concentration using Lowry assay [28]. For in-solution digestion, 5 µg of protein were used for each group of mice. The samples were treated with 10 mM ammonium bicarbonate and the disulfide bonds were reduced with 5 mM dithiothreitol (DTT) in 10 mM ammonium bicarbonate at 60 °C for 1 h. Samples were subsequently alkylated with 15 mM *Iodoacetamide* (IAA) in 10 mM ammonium bicarbonate for 45 min in the dark at room temperature. Protein digestion was done by incubating the samples with 50 ng/µl of sequencing grade trypsin (1:20 trypsin:protein) (Promega, Germany) o/n at 37 °C. Before the injection into the LC–MS/MS, the samples were protonated with 0.1% formic acid.

The tryptic peptides from the digested samples were injected into an Ultimate3000 Nano/Capillary LC System (Thermo Scientific, UK) coupled to a Hybrid quadrupole Q-Tof impact II™ (Bruker Daltonics) equipped with a Nano-captive spray ion source. The peptides were enriched on a µ-Precolumn 300 µm i.d. × 5 mm C18 Pepmap 100, 5 µm, 100 A (Thermo Scientific, UK), separated on a 75 μm I.D. × 15 cm and packed with Acclaim PepMap RSLC C18, two μm, 100 Å, nanoViper (Thermo Scientific, UK). Solvent A and B containing 0.1% formic acid in water and 0.1% formic acid in 80% acetonitrile were supplied on the analytical column. A gradient of 5–55% solvent B was used to elute the peptides at a constant flow rate of 0.30 μl/min for 30 min. Electrospray ionization was carried out at 1.6 kV using the CaptiveSpray. Mass spectra (MS) and MS/MS spectra were obtained in the positive-ion mode over the range (m/z) 150–2200 (Compass 1.9 software, Bruker Daltonics). We performed the LC–MS analysis of each sample in triplicate.

### Bioinformatics and data analysis

The MS data were quantified with MaxQuant 1.6.6.0 using Andromeda search engine to correlate MS/MS spectra to the Uniprot *Mus Musculus* database [29]. Using MaxQuant's standard settings, label-free quantitation was performed. We used trypsin as a digesting enzyme, carbamidomethylation of cysteine as a fixed modification, and the oxidation of methionine and acetylation of the protein N-terminus as variable modifications. We set two miss cleavages as the maximum and a 0.6 Dalton as the main search's mass tolerance. At least one unique peptide with a minimum of 7 amino acids was used for further analysis [30, 31].

The log2 fold change > 1.2 was a cut off for differential expression proteins (DEPs) [31, 32]. The list of differential expression proteins (DEPs) was then inputted to Venn diagrams [33]. The list of up-and down-regulated proteins was then inputted in Panther software for protein classification [34]. Enrichr software was used to analyze enrichment terms from gene ontology (GO) biological processes (https://amp.pharm.mssm.edu/Enrichr/). The functional interaction networks between DEPs and memory-associated molecules were analyzed using the Search Tool for the Retrieval of Interacting Genes/Proteins (STRING) database version 11 (http://string-db.org/cgi/input.pl). The MultiExperiment Viewer (MeV) software [35] was used to produce a heatmap for up-and down-regulated proteins extracted from the GO term "protein phosphorylation."

### Detection of oxidative stress levels in the hippocampus

To measure reactive oxygen species (ROS) levels in the hippocampus, mice were sacrificed, and the brains were isolated 20 min after the contextual test. The procedure was conducted according to a previous study with minor modifications [36]. Briefly, the fixed brains were sectioned by cryostat with 20 µm thickness. Hippocampal sections were then immersed in 1 μmol/l dihydroethidium (DHE) in PBS solution at room temperature for 5 min. The stained sections were washed with 1× PBS three times and cover-slipped. DHE is oxidized by superoxide anion to form ethidium binding to DNA in the nucleus and emits red fluorescence. The images were viewed and taken under a fluorescent microscope (Nikon model #ECLIPSE Ni-E, Japan) with an excitation/emission wavelength of 380/420 nm.

### Immunofluorescence staining

For immunohistochemistry, mice were anesthetized and transcardially perfused using 0.9% saline and 4% paraformaldehyde. Brains were exercised immediately and postfixed with 4% PFA for another 2 days. After that, the brains were washed with 1× PBS three times and then stored in 30% sucrose at 4 °C. After the dehydration period, the brains were embedded in an optimal cutting compound (Sakura Finetek USA, Inc., USA) and stored at − 80 °C until sectioning. Cryopreserved brains were sectioned at 20 µm using cryostat. Brain sections were washed with a washing buffer (1× PBS containing 0.3% Triton X-100) and treated with a permeating buffer (1% Triton X-100 and 2% Tween 20 in 1× PBS) for 30 min. Sections were further blocked with a blocking buffer containing 1% normal goat serum, 0.25% Triton X-100 dissolved in 1× PBS for 1 h. Subsequently, samples were double-stained with polyclonal rabbit anti-GFAP (1:200, Abcam, UK) and monoclonal mouse anti-PRDX6 (1:150, Bethyl laboratories, Inc, USA). The samples were then washed with washing buffer and incubated in secondary antibody: Alexa Fluor 546 anti-mouse and Alexa Fluor 488 anti-rabbit IgG (1:200, ThermoFisher Scientific, USA) for 1 h, followed by washes with PBS, and counterstained with DAPI (1:10,000) for 5 min. The images were obtained by either fluorescent microscope (Nikon model# ECLIPSE Ni-E, Japan) or confocal microscope (Nikon model#C2^+^, Japan).

### Western blot analysis

The mice were sacrificed immediately after the completion of acute immobilization stress. Under trace fear conditioning, hippocampal proteins were extracted at 3 h after training and 20 min after the contextual test. After decapitation, the whole hippocampi were isolated and homogenized in ice-cold RIPA lysis buffer 1× (Millipore, USA) containing protease and phosphatase inhibitors. The protein samples were kept on ice for 30 min before centrifuging at 13,000 rpm for 15 min at 4 °C. The supernatants were collected for further experiments. For non-reducing SDS-PAGE, protein (30 or 45 μg) samples were boiled at 95 °C in 1× sample buffer without reducing agent for 10 min, and samples were cooled for 5 min. Similar to non-reducing conditions, adding reducing agents into protein samples were included to study total PRDX6 and other proteins of interest under reducing condition. The samples were loaded and run on 8% or 10% SDS-PAGE at 80 V in stacking gel and 120 V in resolving gel. The separated proteins were then transferred to a PVDF membrane (0.2 and 0.4 μm pore size) at 30 V overnight. The blots were incubated with anti-NTRK2 (1:1000; Abcam, UK), anti-cPLA2 (1:1000; Santa Cruz, USA), anti-pERK1/2 (1:1000; Cell Signaling, Danvers, MA), anti-total ERK1/2 (1:1000; Cell Signaling, Danvers, MA), anti-PSD95 (1:2000; ThermoFisher, USA), anti-PRDX6 (1:2000; Abcam, UK) or anti-β-actin antibody (1:10,000; Sigma-Aldrich) in TBST containing 0.1% BSA (ThermoFisher, USA) overnight at 4 °C room on a shaker. The next day, the blots were incubated with horseradish peroxidase-conjugated secondary antibody goat anti-mouse IgG (Cell signaling, Danvers, MA) for cPLA2, PSD95, PRDX6, and β-actin and goat anti-rabbit (Santa Cruz Biotechnology, Santa Cruz, CA, USA) for total NTRK2 or TrkB, pERK1/2 and ERK1/2 with the dilution of 1: 10,000 in blocking buffer for 1 h at room temperature. A list of antibodies used in this study was provided in Additional file 2: Table S5. After three washes for 5-min in the TBST buffer, the membranes were developed using ECL (Western lightning^®^ Plus ECL, PerkinElmer Inc, MA, USA) and detected under the UVP Biospectrum 810 imaging system. The band intensities were quantified using ImageJ 1.52a (National Institutes of Health, USA).

### Statistical analysis

Based on previous studies [37–39], we decided to use a sample size from 3 to 20 per group with enough power to see a statistically significant difference. Statistical analysis was performed using SPSS (version 25, IBM Corporation), and the graphs were made using GraphPad Prism version 8. After assessing the normality using the Shapiro–Wilk test, Student's *t*-tests were conducted compared to two independent groups with a normal distribution. In contrast, data that is not normally distributed were assessed by Mann–Withney U-test. One-way ANOVA followed by Bonferroni's post hoc analysis was used for multiple comparisons. For learning ability of TFC and social interaction of three-chamber test, the results were analyzed as mixed-design repeated-measures ANOVA with trials as within-subjects factor and genotypes as a between-subjects factor. The significant interaction was then followed up with the Bonferroni-corrected *t*-test when a significant F-value was determined. All data are presented as mean ± SEM, with statistically significant at *p* < 0.05. Sample sizes are indicated in figure legends.

## Results

### ***Prdx6***^***−/−***^ mice exhibited enhanced fear learning and memory

**Fig. 1:**
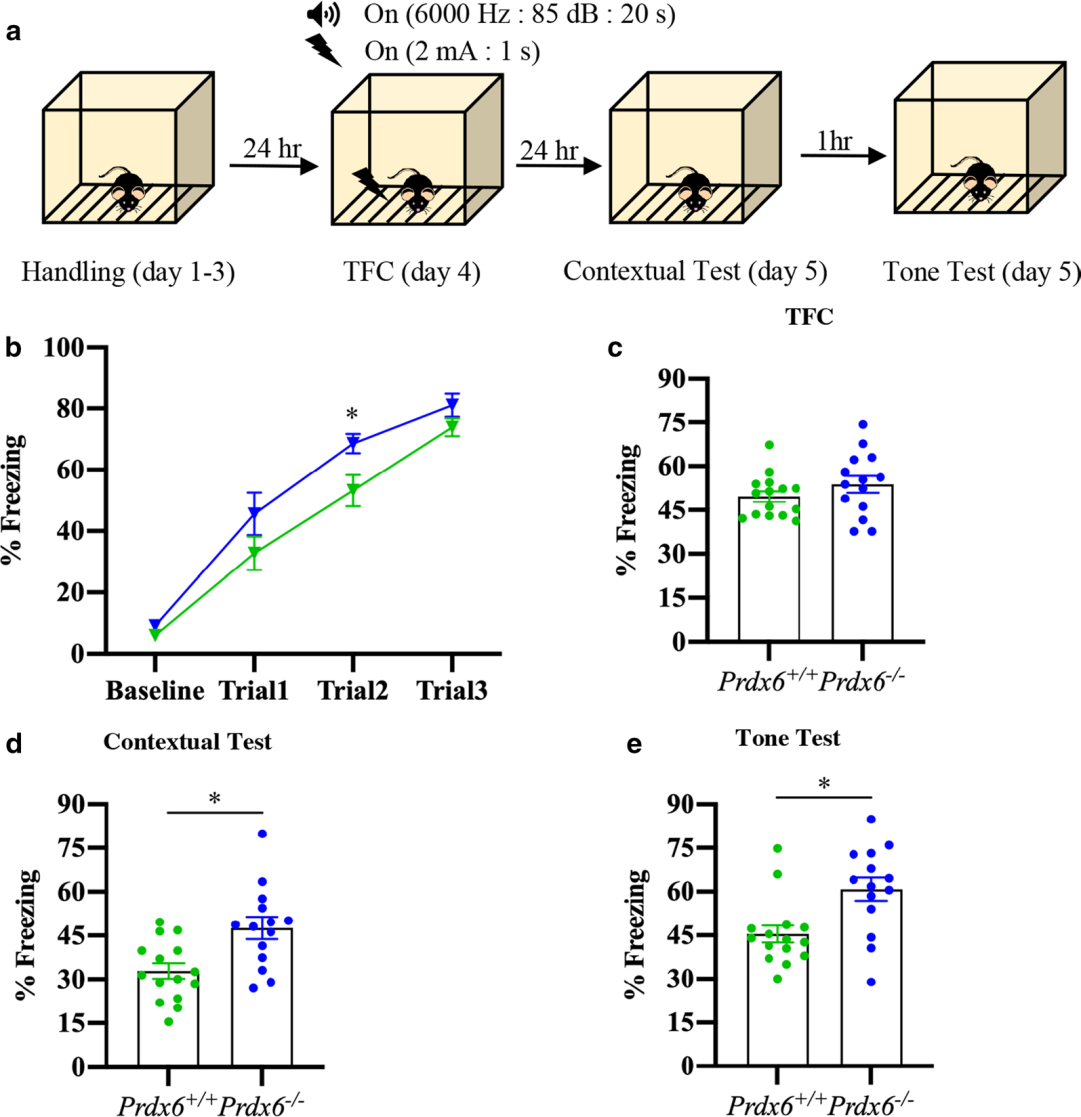
Loss of the *Prdx6* gene caused fast learning and enhanced fear memories. **a** General procedure for trace fear conditioning: Habituation of mice in the chamber was performed for 3 consecutive days. The next day, mice were conditioned with three tone and shock pairs. Contextual fear memories were tested 24 h later, followed by a tone test to evaluate cue fear memory (n = 14/group). **b** The learning curve for baseline and three trials of TFC indicated both groups of mice learnt normally though significant differences appeared in trial 2. **c** Total freezing percentage of *Prdx6*^+/+^ or *Prdx6*^*−*/−^ mice during the training session. **d** Total freezing percentage during the contextual test of mice. **e** Total freezing percentage during the tone test of mice. All data represent the mean ± the SEM. *p < 0.05. *TFC* trace fear conditioning

To identify the function of PRDX6 in fear response, *Prdx6*^*−/−*^ mice underwent trace fear conditioning (TFC) according to the protocol schemed in Fig. 1a. During the first three days, mice were placed in the conditioning chamber and acclimatized to the context for 15 min per day. On day 4, TFC was applied, followed by a contextual test 24 h later. Using mixed design repeated ANOVA, there was no significant effect of the interaction between the genotypes and trials on freezing percentage (*F*_(*2.254*,58.606)_ = 1.042, *p* = 0.366, Fig. 1b) during TFC. The two genotypes exhibited normal learning during the training session, indicated by an increased freezing percentage from baseline to trial 3 as shown by the main effect of trials (*F*_(2.254,58.606)_ = 125.868, *p* = 0.000, Fig. 1b). There was a significant effect of genotypes on freezing percentage during TFC (*F*_(1,26)_ = 6.638, *p* = 0.016, Fig. 1b). Bonferroni-corrected *t*-test revealed significant difference between the two genotypes at trials 2 (*t*_26_ = − 2.580, *p* = 0.016, Fig. 1b). These results suggested that deficiency of PRDX6 leads to fast acquisition of fear memory. No significant difference in total freezing percentage during TFC training (*t*_26_ = − 1.302, *p* = 0.204, Fig. 1c) between the *Prdx6*^+*/*+^ and *Prdx6*^*−/−*^ mice. Interestingly, the *Prdx6*^*−/−*^ mice exhibited a significantly higher freezing response to conditioned context (*t*_26_ = − 2.985, *p* = 0.006, Fig. 1d) and cue (*t*_26_ = − 2.956, *p* = 0.007, Fig. 1e) than the *Prdx6*^+*/*+^ mice suggesting the impact of the *Prdx6* gene on the regulation of contextual and cued fear memories.

### Lentivirus containing mouse PRDX6 (LV-mPRDX6) attenuated contextual fear memory of *Prdx6*^*−/−*^ mice

**Fig. 2:**
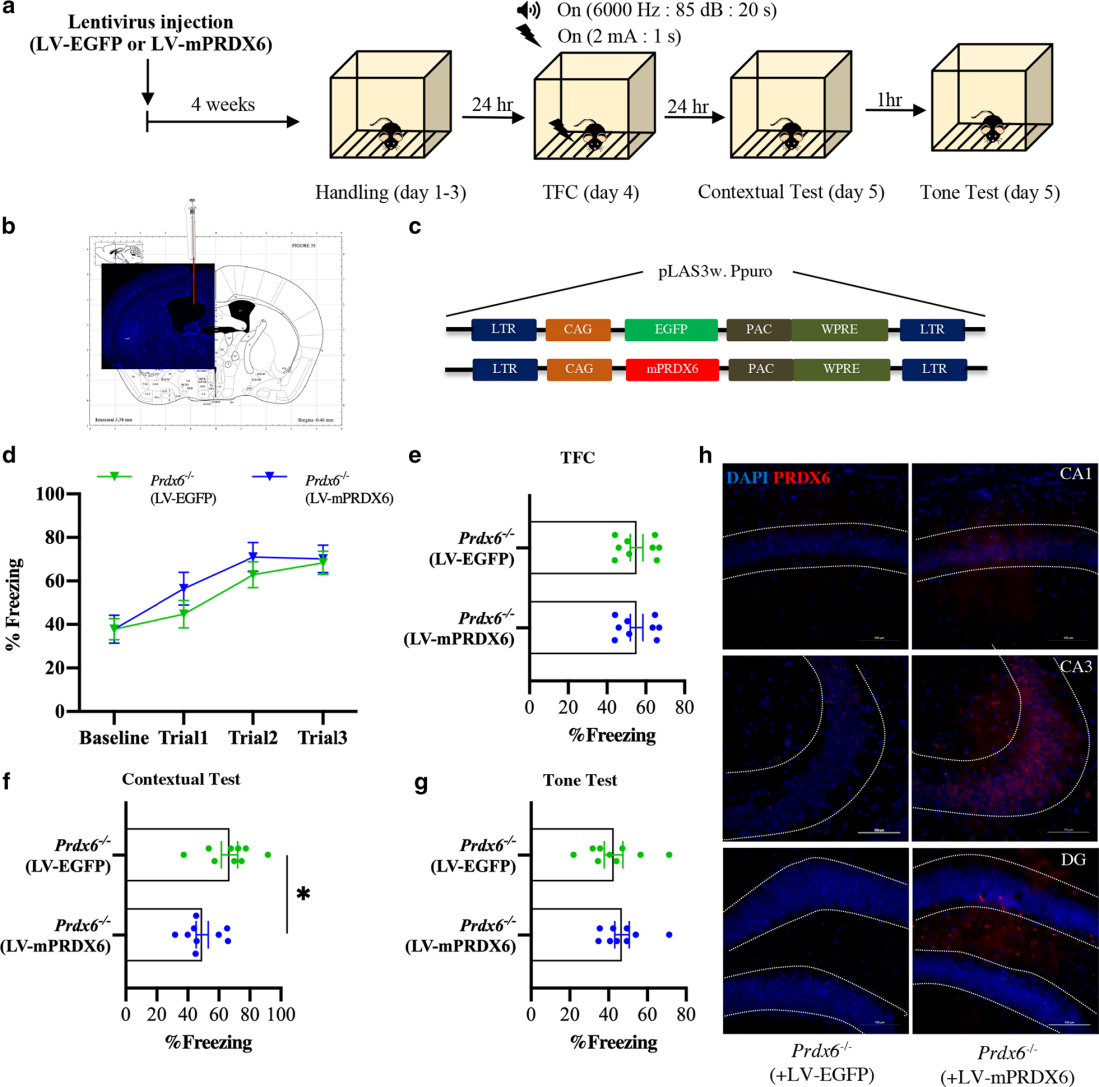
Intraventricular injection of mouse PRDX6 (mPRDX6) lentiviruses attenuated enhanced contextual fear memory of *Prdx6*^*−*/−^ mice. **a** The procedure for the overexpression study. Mice were injected with lentivirus and housed for 2 weeks before performing trace fear conditioning. **b** Representative image of cannula tip position (red) in the right lateral ventricle. **c** The schematic lentivirus construct pLAS3w. *Ppuro* contains either EGFP or mPRDX6. **d** The learning curve of baseline and after each tone-shock pair (n = 10/group). **e** Total freezing percentage of *Prdx6*^+/+^ and *Prdx6*^*−*/−^ mice during the training session. **f** Total freezing percentage during the contextual test of mice. **g** Total freezing percentage of the mice during the tone test. **h** mPRDX6 expression across the different subregions in the hippocampus of *Prdx6*^*−*/−^ mice**.** All data represent the mean ± the SEM. *p < 0.05. *TFC* trace fear conditioning, *LV-mPRDX6* lentivirus containing mouse PRDX6

To further confirm the role of PRDX6 in the expression of fear memory, the gain-of-function study was conducted by intracerebroventricularly injecting LV-mPRDX6 into the lateral ventricle near the hippocampal region of *Prdx6*^*−/−*^ mice. The mice were then subjected to TFC 4 weeks after the injection (Fig. 2a). Figure 2b and c illustrate the site of injection and lentiviral construct, respectively. There was no effect of group on learning ability as shown in Fig. 2d (*F*_(*1*,16)_ = 0.551, *p* = 0.469; *Prdx6*^*−/−*^ mice with LV-EGFP *vs* LV-mPRDX6). Both groups displayed normal learning during training sessions indicating an increased freezing percentage from baseline to trial 3 as shown by the main effect of trials (*F*_(3,48)_ = 26.691, *p* = 0.000, Fig. 2d). During the training session, the total freezing percentage was similar between the two groups (*t*_16_ = − 0.654, *p* = 0.522, Fig. 2e), indicating that the injection of LV-mPRDX6 did not affect the learning ability of the *Prdx6*^*−/−*^ mice. Importantly, lentiviral injection of mPRDX6 successfully reduced enhanced contextual fear response of the *Prdx6*^*−/−*^ mice (*t*_16_ = 2.698, *p* = 0.016, Fig. 2f). However, re-expression of mPRDX6 failed to rescue cue fear memory (*t*_16_ = − 0.700, *p* = 0.494, Fig. 2g). Fluorescent images demonstrated the expression of mPRDX6 (Fig. 2h) and EGFP (Additional file 1: Fig. S2a, b) in three hippocampal regions, including the CA1, CA3, and DG after the completion of the tone test. We also detected the expression of mPRDX6 in the amygdala and prefrontal cortex (Fig. 2h and Additional file 1: Fig. S3a, b). These results suggest that hippocampal PRDX6 is involved in regulating fear expression, at least for contextual fear memory.

### Deletion of the *Prdx6* gene caused hyperlocomotion without affecting social exploration and recognition

**Fig. 3:**
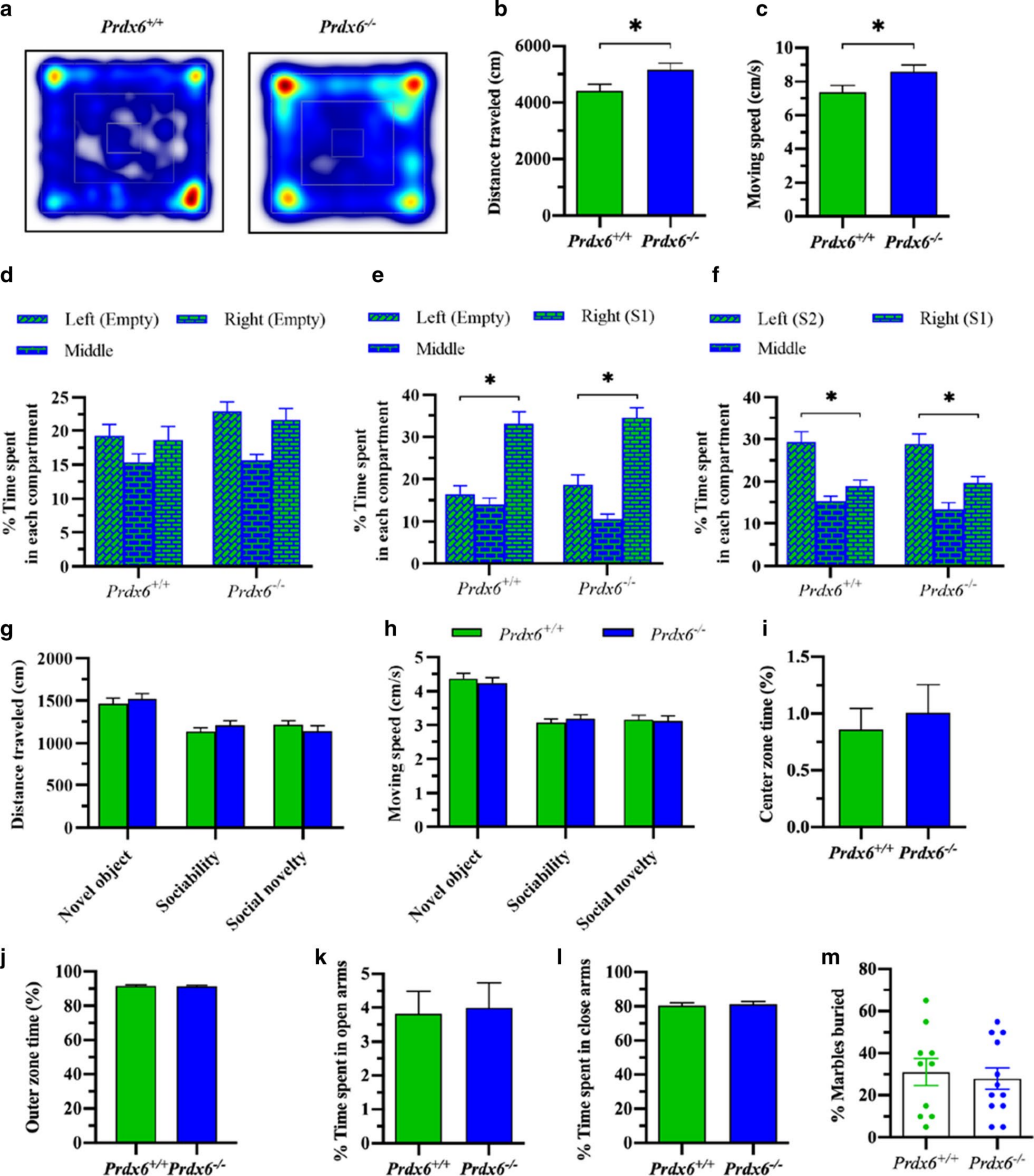
Increased locomotor function, but normal anxiety-like behavior, exploration, sociability, and social novelty in *Prdx6*^*−/−*^ mice. **a** Heatmaps during 10 min of exploration in an open field chamber. **b** Quantification data of distance traveled for 10 min (n = 15–18/group, Student’s *t*-test). **c** The mean moving speed (cm/s) of the mice introduced in the open field test*.* **d** Time spent on each side of the chamber containing empty wire cups (novel object) (n = 17/group). **e** Time spent on each side of the chamber containing a stranger mouse 1 (S1) or empty wire cup. **f** Time spent on each side of the chamber containing familiar mouse 1 (S1) or novel mouse (S2). **g** The mean distance traveled during three trials of the task. **h** The mean moving speed during three trials of a task. **i** The mean percentage of center zone time (n = 15–18/group, Student's t-test). **j** The mean percentage of outer zone time. **k** Percent time spent in open arms (n = 18–20/group). **l** Percent time spent in close arms. **m** Percent marbles buried in the marble-burying test. All data represent the mean ± the SEM. *p < 0.05

The heatmap during 10 min of exploration in the open field chamber was presented in Fig. 3a. The total distance traveled (*t*_31_ = − 2.191, *p* = 0.036, Fig. 3b) and moving speed (*t*_31_ = − 2.197, *p* = 0.036, Fig. 3c) of the *Prdx6*^*−/−*^ mice were significantly higher than those of the *Prdx6*^+*/*+^ mice. An open field test indicated that *Prdx6*^*−/−*^ mice exhibited hyperlocomotion compared with *Prdx6*^+*/*+^ mice; hence higher freezing response to context did not result from reduced locomotion. We then assess object exploration, sociability and social novelty behaviors of the *Prdx6*^−/−^ mice using a three-chamber apparatus [40]. For the novel object exploration test (trial 1), both genotypes demonstrated a significant preference for exploring empty cups, and no significant genotype effect was observed (side: *F*_(1.604,52.935)_ = 46.642, *p* = 0.000; genotype: *F*_(1,33)_ = 0.003, *p* = 0.958; genotype × side: *F*_(1.604,52.935)_ = 0.794, *p* = 0.432, Fig. 3d). The stranger mouse 1 (S1) was placed in the right compartment within an inverted wire cup for the sociability test. Both genotypes demonstrated a significant preference for exploring stranger mouse 1 and no significant genotype effect was observed (side: *F*_(2,66)_ = 28.869, *p* = 0.000; genotype: *F*_(1,33)_ = 0.232, *p* = 0.633; genotype × side: *F*_(2,66)_ = 0.118, *p* = 0.889, Fig. 3e). In the social novelty preference test, the interaction duration with the novel mouse (S2) appeared to be normal since the *Prdx6*^−/−^ mice spent similar time with the novel mouse compared to wild-type group (*F*_(1,20)_ = 0.000; *p* = 0.991, Fig. 3f). Both genotypes stayed with the novel mouse longer than the familiar mouse (S1) (*F*_(1.495,29.895)_ = 11.089; *p* = 0.001, Fig. 3f), representing the normal response of social novelty. In each test, no significant difference in locomotor activity was recorded, measured by equal distance traveled (*t*_38_ = − 1.056, *p* = 0.297, Fig. 3g) and moving speed (*t*_38_ = 0.340, *p* = 0.736, Fig. 3h) between the two genotypes.

### Normal anxiety-like behavior and hypervigilance in *Prdx6*^*−/−*^ mice

We next investigated anxiety response and hypervigilance in *Prdx6*^−/−^ mice using an open field, elevated plus-maze, and marble burying tests, respectively. We observed equal time spent in the center (*t*_31_ = − 0.493, *p* = 0.632, Fig. 3i) and outer zone (*t*_31_ = 0.235, *p* = 0.816, Fig. 3j) in an open-field chamber between the two genotypes. The *Prdx6*^−/−^ mice showed similar results in elevated plus-maze as controls indicated by equal time spent in open arms (U = 171, Z = − 0.263, *p* = 0.792, Fig. 3k) and close arms (*t*_36_ = − 0.180, *p* = 0.858, Fig. 3l) indicating normal anxiety-like behavior in *Prdx6*^−/−^ mice. Performing the marble burying test, we observed no significant difference in the percentage of buried marbles between the two genotypes (*t*_20_ = 0.378, *p* = 0.709, Fig. 3m). This result demonstrated that deletion of the *Prdx6* gene did not cause hypervigilance.

### Proteomic analysis for total hippocampal proteins extracted during the contextual memory retrieval stage

**Fig. 4:**
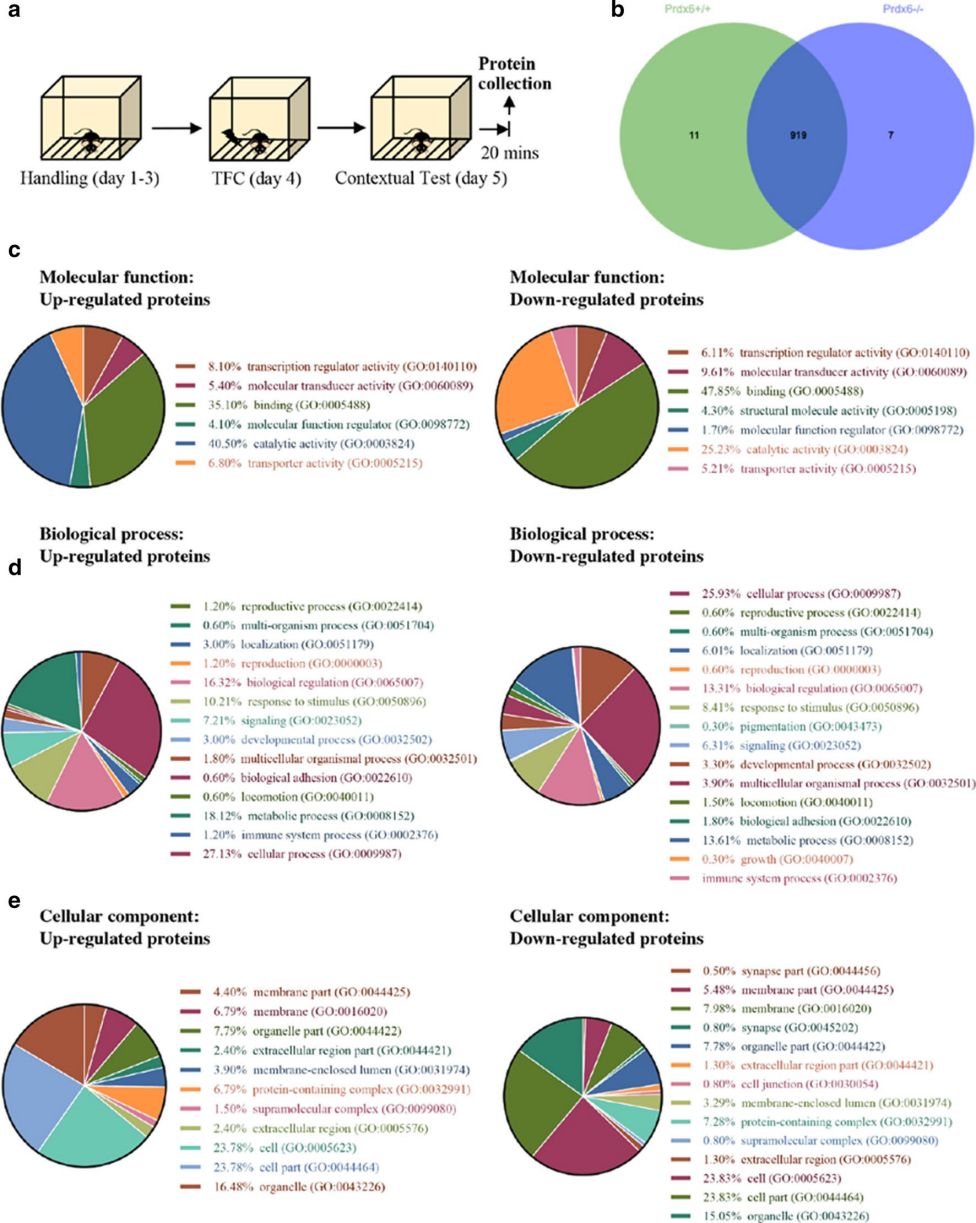
Three functional classifications of the proteins in the hippocampus of mice re-exposed to conditioned chambers. **a** The schematic diagram of trace fear conditioning and protein collection (n = 3/group). **b** Venn diagram defining the difference in protein expressions between *Prdx6*^*−*/−^ and *Prdx6*^+*/*+^ mice. The 125 upregulated proteins and 130 down-regulated proteins affected by the contextual test were classified into three functional classifications: **c** molecular function, **d** biological process, and **e** cellular component

To understand what hippocampal proteins are involved in the retrieval process of contextual memory, we conducted a proteomic analysis for total hippocampal proteins collected during the retrieval stage of TFC (Fig. 4a). Liquid chromatography–tandem mass spectrometry (LC/MS–MS) provided a total of 937 proteins that differentially expressed in the hippocampus of *Prdx6*^*−/−*^ and *Prdx6*^+*/*+^ mice. The top 20 up- and down-regulated differential expression proteins (DEPs) was provided in Additional file 2: Table S1. All proteins on the list from both genotypes were plotted in Venn diagrams based on their expressions (Fig. 4b). There were 11 proteins specifically expressed in *Prdx6*^+*/*+^ mice, 7 proteins expressed only in *Prdx6*^*−/−*^ mice, and 919 proteins expressed in both genotypes. Using Panther software, the differential expression proteins (DEPs) were classified into three gene ontologies (GO): molecular function, biological process, and cellular component. According to molecular functions, the most overrepresented groups were catalytic activity (40.50% up- and 25.23% down-regulated proteins, Fig. 4c) and binding (35.10% up- and 47.85% down-regulated proteins). In the GO biological process, the main biological processes of DEPs were cellular processes (27.13% up- and 25.93% down-regulated proteins, Fig. 4d) and metabolic processes (16.32% up- and 3.61% down-regulated proteins). The analysis of cellular components indicated that cell (the plasma membrane and any external encapsulating structures; 23.78% up- and 23.83% down-regulated proteins, Fig. 4e) and cell part (any constituent part of a cell; 23.78% up- and 23.83% down-regulated proteins) were the main cellular components of DEPs.

### PRDX6 regulates fear memory retrieval via the MAPK signaling pathway

Oxidative stress is known to be involved in the modulation of fear memory [41]. To measure the oxidative status in the hippocampus of *Prdx6*^*−/−*^ mice during memory retrieval, we performed dihydroethidium (DHE) staining. The brains were collected 20 min after the contextual test (Additional file 1: Fig. S4a). Additional file 1: Figure S4b shows the ethidium fluorescence of DHE. Quantitative analysis showed no significant difference in the DHE-positive density was found between genotypes in both CA1 (*t*_4_ = − 0.508, *p* = 0.638, Additional file 1: Fig. S4c) and CA3 (*t*_4_ = − 0.060, *p* = 0.0.955, Additional file 1: Fig. S4d) subregions of the hippocampus. The results demonstrated that PRDX6 might not regulate fear response by controlling cellular oxidation. Moreover, it led us to question whether PRDX6 directly modulates the cellular signaling cascade to control fear memory expression.

**Fig. 5:**
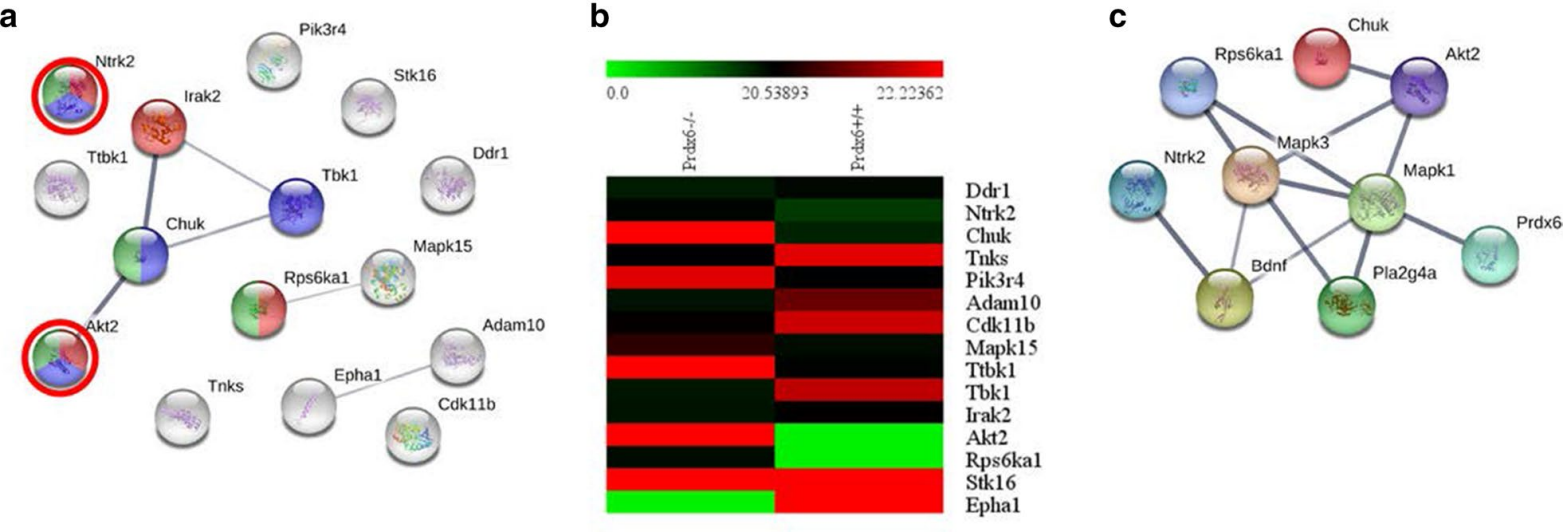
Proteomic analysis revealed differential expression proteins (DEPs) MAPK and Ras signaling pathways in the hippocampus of *Prdx6*^*−/−*^ mice during retrieval of contextual fear memory. **a** Functional protein–protein interaction networks of 15 proteins related to GO term "protein phosphorylation" (GO:0006468). The significant nodes were labeled in red for Neurotrophin signaling pathway (FDR 0.00012), blue for the Ras signaling pathway (FDR 0.00073), and green for the MAPK signaling pathway (FRD 0.0011). **b** Heatmap of 15 proteins in GO term "protein phosphorylation" (GO:0006468) with 8 upregulated proteins and 7 down-regulated proteins in *Prdx6*^*−/−*^ mice. **c** STRING showed a predicted functional protein–protein interaction network of proteins in the KEGG pathway termed "MAP kinase signaling pathway" with memory-associated proteins and PRDX6. The significant nodes were labeled in red for the MAPK signaling pathway (FDR 1.25e−12) and green for the Ras signaling pathway (FDR 3.33e−11)

**Fig. 6:**
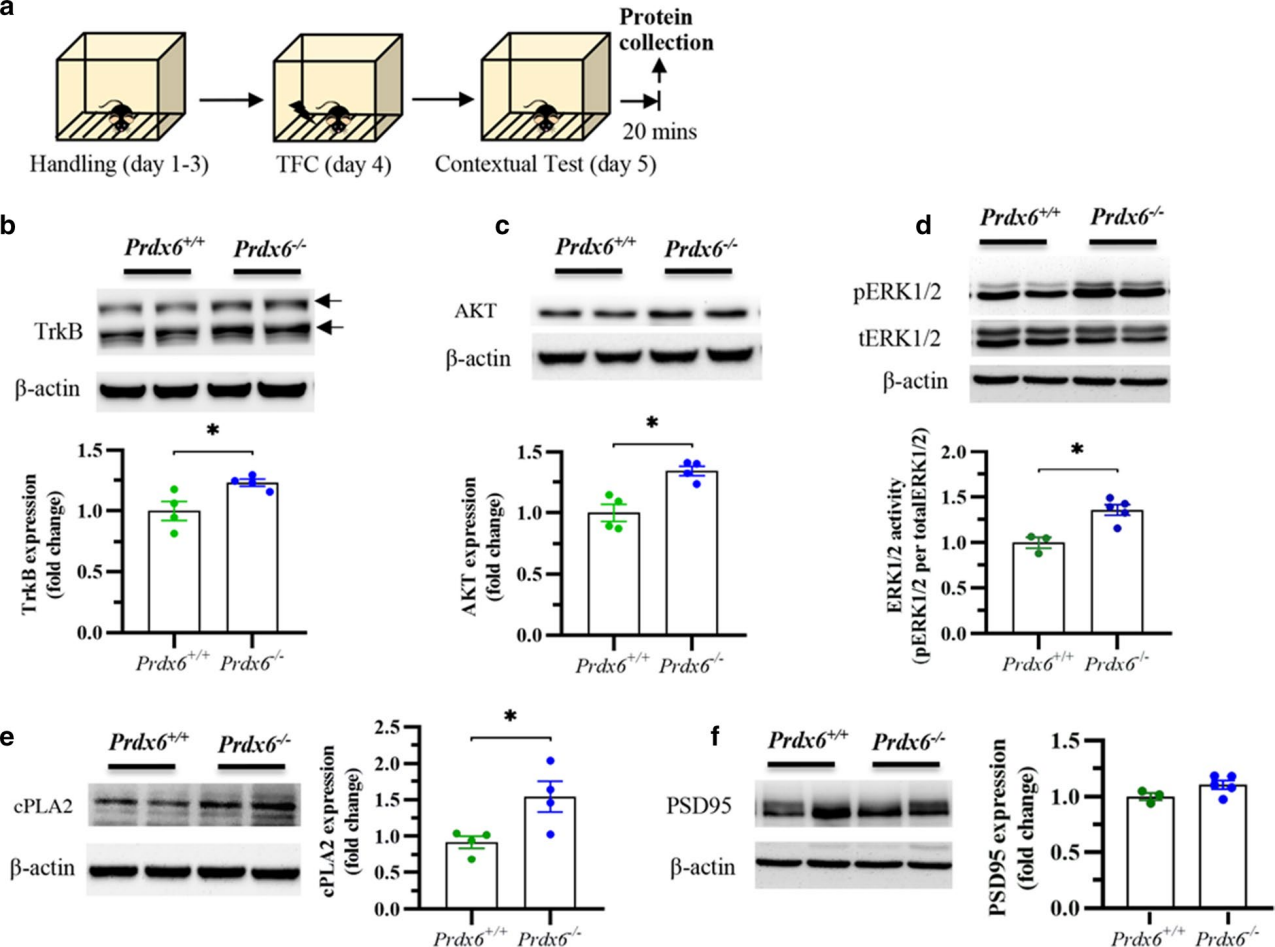
Activation of MAPK signaling pathways in the hippocampus of *Prdx6*^*−/−*^ mice during retrieval of contextual fear memory. **a** Hippocampal protein samples were collected 20 min after the contextual test for the retrieval process to validate proteins in the MAPK signaling pathway (mmu04010), including TrkB (NTRK2), AKT, pERK1/2, and cPLA2. (**b**–**f**; upper panels) Immunoblots of TrkB, AKT, pERK1/2, tERK1/2, cPLA2, PSD95, and β-actin expression in the hippocampus during memory retrieval. (**b**–**f**; lower panels) Quantification data for the expression levels of TrkB, AKT, phosphorylated ERK1/2, cPLA2, and PSD95 in the hippocampi of mice (n = 3–5/group). All data represent the mean ± the SEM. *p < 0.05. *TrkB* tyrosine receptor kinase B, *AKT or PKB* Protein kinase B, *PSD95* postsynaptic protein density 95

To delineate the molecular pathways responsible for the enhanced fear response of the *Prdx6*^*−/−*^ mice, Enrichr software was then conducted to identify the enriched biological process of the DEPs (cut-off 1.2 fold change) in *Prdx6*^*−/−*^ mice. The protein phosphorylation (GO: 0006468) (*p* = 0.0056) was one of the significant enrichment terms from the GO biological process of DEPs in *Prdx6*^*−/−*^ mice (Additional file 2: Table S2; the top 25 enrichment terms from GO biological process). We next extracted 15 DEPs from the GO term "protein phosphorylation" (GO: 0006468) (Additional file 2: Table S3). The 8 proteins that were upregulated and 7 proteins that were down-regulated in *Prdx6*^*−/−*^ mice were input to STRING software to obtain the networks of protein–protein interaction involved in memory processes (Fig. 5a, b). The significant nodes of DEPs were identified according to the Kyoto Encyclopedia of Genes and Genomes (KEGG) pathway database (Additional file 2: Table S4). The DEPs of *Prdx6*^*−/−*^ mice were strongly associated with the neurotrophin signaling pathway (false discovery rate or FDR = 0.00012), Ras signaling pathway (FDR = 0.00073), and MAPK signaling pathway (FDR = 0.0011). These pathways are well known to fear memory consolidation and retrieval [42, 43]. To study the molecular changes that participate in regulating contextual fear memory retrieval of *Prdx6*^*−/−*^ mice, we extracted DEPs from the MAPK signaling pathway, including AKT2, CHUK, NTRK2, and RPS6KA1. We created new networks, including MAPK1, MAPK3, BDNF, cPLA2, and PRDX6, using STRING software (Fig. 5c). During retrieval (Fig. 6a), western blot analysis was performed to confirm the expression of the key proteins from the network, including NTRK2, AKT, ERK1/2, and cPLA2, during retrieval of contextual memory. Significant upregulation of NTRK2 (or TrkB) (*t*_6_ = − 2.798, *p* = 0.031, Fig. 6b), AKT (*t*_6_ = − 4.242, *p* = 0.005, Fig. 6c), ERK1/2 phosphorylation (*t*_5_ = − 5.336, *p* = 0.003, Fig. 6d) and cPLA2 (*t*_6_ = − 2.761, *p* = 0.033, Fig. 6e) were recorded in the hippocampus of *Prdx6*^*−/−*^ mice after a contextual test. Postsynaptic density protein 95 (PSD95), a postsynaptic marker, was also detected and no significant difference was recorded (*t*_*6*_ = − 1.843, *p* = 0.115, Fig. 6f) in *Prdx6*^*−/−*^ mice. These results demonstrated the correlation of the MAPK pathway with PRDX6 to regulate fear memory retrieval.

### Co-localization of PRDX6 with the astrocytic marker, GFAP, in the hippocampus

**Fig. 7:**
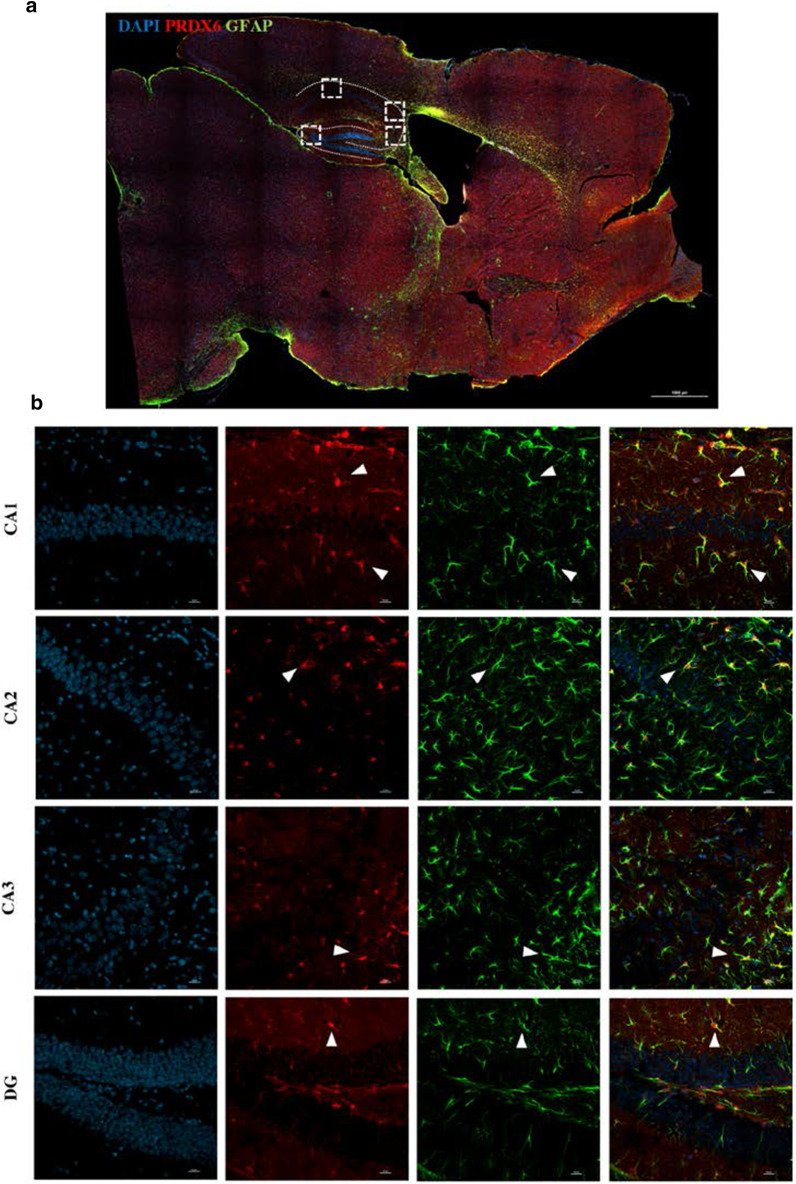
PRDX6 protein is highly expressed in the astrocytes throughout the hippocampus. **a** Sagittal section of the brain showing colocalization of PRDX6 (red) with GFAP (green), scale bar 1000 μm. **b** Confocal images of PRDX6-GFAP colocalization in the astrocytes of CA1, CA2, CA3, and DG subregions of the hippocampus, scale bar 20 μm

Since we focused on identifying the function of PRDX6 in the regulation of fear memory, we then confirmed the distribution of PRDX6 in three brain regions primarily involved in fear memory formation—the hippocampus [13], amygdala [11], and prefrontal cortex [44]. Previous studies report that PRDX6 is highly expressed in the astrocytes [11] under various conditions but not known in TFC. We thus performed double staining with anti-PRDX6 and anti-GFAP (astrocyte marker) antibodies to examine whether PRX6 is also expressed in astrocytes after TFC. Our results demonstrated that PRDX6 is expressed in the hippocampal astrocytes within the CA1, CA2, CA3, and DG (Fig. 7a, b). We also recorded the expression of PRDX6 in the amygdala (Additional file 1: Fig. S5a, b) and prefrontal cortex (Additional file 1: Fig. S6a, b).

## Discussion

The present study reports that the loss of the *Prdx6* gene in the brain led to enhanced trace fear memory to context. The intracerebroventricular injection (i.c.v) of LV-mPRDX6 could reverse the enhanced contextual fear response, a hippocampal-dependent memory, of *Prdx6*^−/−^ mice. We confirmed that the observed effect was attributable to PRDX6. Proteomic and western blot analysis revealed that mitogen-activated protein kinase (MAPK) signaling pathways were highly activated in the hippocampi of *Prdx6*^−/−^ mice during the expression of contextual fear memory. These results suggest that PRDX6 plays a critical role in the regulation of fear memory expression.

In humans, the feeling of intense fear has been defined using the Diagnostic and Statistical Manual of Mental Disorders, fifth edition (DSM-V) as one primary symptom of PTSD [45, 46]. Three brain regions, the hippocampus, amygdala, and prefrontal cortex, are important for fear memory formation [6]. Here we demonstrated that PRDX6 is expressed in the astrocytes of the amygdala (Additional file 1: Fig. S5), prefrontal cortex (Additional file 1: Fig. S6), and hippocampus (Fig. 7a, b) after TFC. The activation of hippocampal astrocytes plays a crucial role in synaptic plasticity and contextual fear memory [47, 48]. It is known that PRDX6 can modulate astrocyte activation [49, 50]. Whether PRDX6 may regulate the activation of astrocytes during the synaptic process and memory formation requires further study to verify.

In the present study, we used systemic *Prdx6* knockout strain, which lacks PRDX6 in the whole brain [23], for trace fear conditioning (TFC). Since intracerebroventricular injection of mouse PRDX6 lentivirus reduced contextual fear memory, we thus focused on identifying the function of PRDX6 in the required brain region—the hippocampus [51, 52]. We found that PRDX6 is colocalized with an astrocytic marker, EGFP, within the hippocampus. Although high expression level of PRDX6 in astrocytes was confirmed, its expression in other cell types was not examined in the present study. Given that PRDX6 expression in different cell types would affect animal behavior, designing a construct containing a neuron or oligodendrocyte specific promotor may help identify related molecular and cellular mechanisms regarding PRDX6′s function in memory formation.

Our results also demonstrate that the *Prdx6*^*−/−*^ mice displayed hyperlocomotion activity. This phenotype confirms that enhanced freezing behavior exhibited in *Prdx6*^−/−^ mice was attributable to the lack of PRDX6, not reduced locomotor activity. Anxiety-like, motivation, and exploration behaviors may also affect response to fear conditioning [53, 54]. These behaviors are normal in *Prdx6*^*−/−*^ mice, indicating loss of *PRDX6* does not cause these phenotypes. This series of behavior tests suggest that the ablation of *Prdx6* is specifically responsible for the enhanced fear memory.

The excessive fear expression to TFC exhibited by *Prdx6*^−/−^ mice was also observed in activating transcription factor 3 (*Atf3*) deficient mice [17]. ATF3 is a leucine zipper-containing (bZIP) transcription factor-induced upon stress [55]. Using a computer-based search program (Alggen Promo software, version 8.3), we found that the promoter region of the *Prdx6* gene contains binding sites for activating transcription factor 3 (ATF3). Moreover, proteomic analysis (Additional file 2: Table S1) reveals that expression of gelsolin (GSN), an actin-severing protein essential for synaptic plasticity [56], is reduced in the hippocampus of *Prdx6*^*−/−*^ mice. This phenomenon is also recorded in the *Atf3*^*−/−*^ mice after TFC [17]. Besides, in rats subjected to predator-scent-stress (PSS), a PTSD-like model, gelsolin (*Gsn*) expression levels were also downregulated [57]. We thus speculate that ATF3, PRDX6, and GSN may participate in the same or related pathways for the regulation of fear memory. Further experiments are necessary to verify their relationship.

Inhibition of the memory retrieval process is proved to attenuate excessive fear response [42]. Stress and stress hormone, glucocorticoid (GC) may positively or negatively affect fear response involving stress coping mechanisms [58, 59]. PRDX6 can be regulated by dexamethasone, a glucocorticoid analog suggesting a possible role of PRDX6 in stress coping mechanisms, including fear response [60]. Previous studies have shown the physiological and pathological role of reactive oxygen species (ROS) in fear response [61], and the hippocampal pyramidal neurons of the CA1 and CA3 subregions related to fear memory retrieval are more vulnerable to oxidative stress [51, 62, 63]. During contextual memory retrieval, ROS level in the hippocampal CA1 and CA3 regions of the *Prdx6*^*−/−*^ mice remained similar as wild-type mice (Additional file 1: Fig. S4), indicating that PRDX6 did not regulate the expression of contextual fear memory through modulating ROS level in the hippocampus.

The proteomic and western blot analysis revealed that upregulation of several proteins (Additional file 2: Table S1) involved in the MAPK signaling pathway in the hippocampus of *Prdx6*^*−/−*^ mice during the retrieval stage of contextual fear memory. It is known that ongoing protein synthesis is required for maintaining GluA1-AMPA receptors at the synapses for cue memory retrieval [64]. Tropomyosin receptor kinase B (TrkB) and its downstream molecules (AKT and ERK1/2) participate in the regulation of production and trafficking of GluA1-AMPA receptors [65]. A previous study revealed immediate upregulation of total TrkB after a probe test [66]. Another study showed upregulation of total AKT in the basolateral amygdala (BLA) 15 min after reexposure to conditioned context [67]. We also found immediate upregulation of these proteins in the hippocampi of *Prdx6*^*−/−*^ mice. Further studies are necessary to verify whether upregulation of these proteins results from local protein synthesis during contextual fear memory retrieval. One previous research has shown that PRDX6 participates in the modulation of ERK1/2 activity in the lung [68]. Another study revealed that inhibition of ERK1/2 before a memory test blocks contextual fear memory retrieval [42]. These studies suggest that hyperphosphorylation of ERK1/2 is associated with enhanced contextual fear memory in the *Prdx6*^*−/−*^ mice. Among differential expression proteins listed on Additional file 2: Table S1, total TrkB is highly expressed in the hippocampus of *Prdx6*^*−/−*^ mice. This neurotrophin receptor is encoded by the neurotrophic receptor tyrosine kinase 2 (*Ntrk2*) gene and plays an important role in neuronal plasticity and fear memory [69]. Piazza and colleagues reported that the mice administered with stress hormone GC exhibited enhanced fear response via the activation TrkB/MAPK pathway [58]. Thus, hyperactivation of ERK1/2 in the absence of PRDX6 may be correlated with increased TrkB level.

Interestingly, we also observed upregulation of cytosolic phospholipase A2 (cPLA2), a downstream target of ERK1/2 [70], in the *Prdx6*^*−/−*^ mice, which may be the compensation effect for the functional loss of aiPLA2-PRDX6 [71]. This increased cPLA2 level may promote contextual fear memory retrieval in *Prdx6*^*−/−*^ mice, since blocking cPLA2 activity before memory test suppresses memory retrieval [72]. TrkB signaling can also activate phosphoinositide 3-kinase (PI3K) and protein kinase B (AKT) [73, 74]. Blocking of PI3K reduced activation of ERK1/2 and AKT, in turn, impaired fear memory retrieval [73]. These pieces of data suggest that activation of TrkB signaling and its downstream molecules-ERK1/2, cPLA2, and AKT in the hippocampus may help enhance retrieval of fear memory in *Prdx6*^−/−^ mice. Other brain regions may also be responsible for the *Prdx6*^*−/−*^ mice's enhanced contextual fear response, particularly the amygdala, but the protein changes were not examined in the present study. A further experiment is worth pursuing the amygdala’s significance in regarding this phenotype of the *Prdx6*^*−/−*^ mice*.*

In conclusion, this study is the first to report PRDX6′s function in negative regulation of contextual fear memory along with hyperactivation of the MAPK pathway in the hippocampus during the retrieval stage of contextual memory. The results obtained from this study reveal the physiological role of PRDX6 in memory formation and help better understand the mechanism underlying homeostatic fear regulation. It also suggests that PRDX6 may be a potential drug target for treating fear-dysregulated disorders like PTSD.

## Supplementary Information

**Additional file 1: Figure S1–S6.** The characteristics of *Prdx6*^*−/−*^ mice (**Fig. S1**). Expression of EGFP in the hippocampus of *Prdx6*^*−/−*^ mice (**Fig. S2**). Immunostaining of mPRDX6 expressed in the amygdala and prefrontal cortex (**Fig. S3**). The level of reactive oxygen species (ROS) measured by superoxide-sensitive DHE staining in hippocampal CA1 and CA3 regions of *Prdx6*^*+/+*^ and *Prdx6*^*−/−*^ mice (**Fig. S4**). Co-localization of PRDX6 with GFAP in the amygdala (**Fig. S5**) and prefrontal cortex (**Fig. S6**).

**Additional file 2: Table S1–S5.** This file contains the list of differential expression proteins (DEPs) in the hippocampus of *Prdx6*^+*/*+^ and *Prdx6*^*−/−*^ mice after a contextual test (**Table S1**). First 25th enrich terms of GO biological process of differential expression proteins (DEPs) (**Table S2**). Up- and down-regulated enrich proteins differentially expressed relative to GO biological process termed MAPK signaling pathway during contextual memory retrieval in *Prdx6*^*−/−*^ mice (**Table S3**). List of KEGG pathways for GO biological process termed "protein phosphorylation" (GO:0006468) of up-and down-regulated proteins (**Table S4**). And the details of antibodies and vectors used in this study (**Table S5**).

## Data Availability

The data that support the findings of this study are available from the corresponding author upon reasonable request.

## Abbreviations

aiPLA2: Acidic calcium-independent phospholipase A2
ATF3: Activating transcription factor 3
CA1: Cornu ammonis 1
CA3: Cornu ammonis 3
cPLA2: Cytosolic phospholipase A2
DEPs: Differential expression proteins
DG: Dentate gyrus
DHE: Dihydroethidium
DSM-V: Diagnostic and Statistical Manual of Mental Disorders, fifth edition
ERK1/2: Extracellular signal-regulated protein kinases 1 and 2
FDR: False discovery rate
GC: Glucocorticoid
GO: Gene ontology
GPx: Glutathione peroxidase
LC/MS–MS: Liquid chromatography–tandem mass spectrometry
LPCAT: Lysophosphatidylcholine acyltransferase
LV-mPRDX6: Lentivirus-carrying mouse PRDX6
MAPK: Mitogen-activated protein kinase
NTRK2: Neurotrophic receptor tyrosine kinase 2
PI3K: Phosphoinositide 3-kinase
PRDX6: Peroxiredoxin 6
PTSD: Post-traumatic stress disorder
ROS: Reactive oxygen species
