# Supplementary material for: Enhanced contextual fear memory in peroxiredoxin 6 knockout mice is associated with hyperactivation of MAPK signaling pathway
Source: Mol Brain. 2021 Feb 25;14:42. doi: 10.1186/s13041-021-00754-1 (PMC7908735; doi:10.1186/s13041-021-00754-1)
Supplement: Supplementary file 1 — Additional file 1: Figure S1–S6. The characteristics of Prdx6−/− mice (Fig. S1). Expression of EGFP in the hippocampus of Prdx6−/− mice (Fig. S2). Immunostaining of mPRDX6 expressed in the amygdala and prefrontal cortex (Fig. S3). The level of reactive oxygen species (ROS) measured by superoxide-sensitive DHE staining in hippocampal CA1 and CA3 regions of Prdx6+/+ and Prdx6−/− mice (Fig. S4). Co-localization of PRDX6 with GFAP in the amygdala (Fig. S5) and prefrontal cortex (Fig. S6). [file 13041_2021_754_MOESM1_ESM.docx]

**Supplementary figures**

**Enhanced contextual fear memory of peroxiredoxin 6 knockout mice is associated with hyperactivation of MAPK signaling pathway**

Sarayut Phasuk^1,2^, Tanita Pairojana^1^, Pavithra Suresh^1^, Chee-Hing Yang^3^, Sittiruk Roytrakul^4^, Shun-Ping Huang^6^, Chien-Chang Chen^5^, Narawut Pakaprot^2^, Supin Chompoopong^7^, Sutisa Nudmamud-Thanoi^8,9,^ and Ingrid Y. Liu^1, *^

**
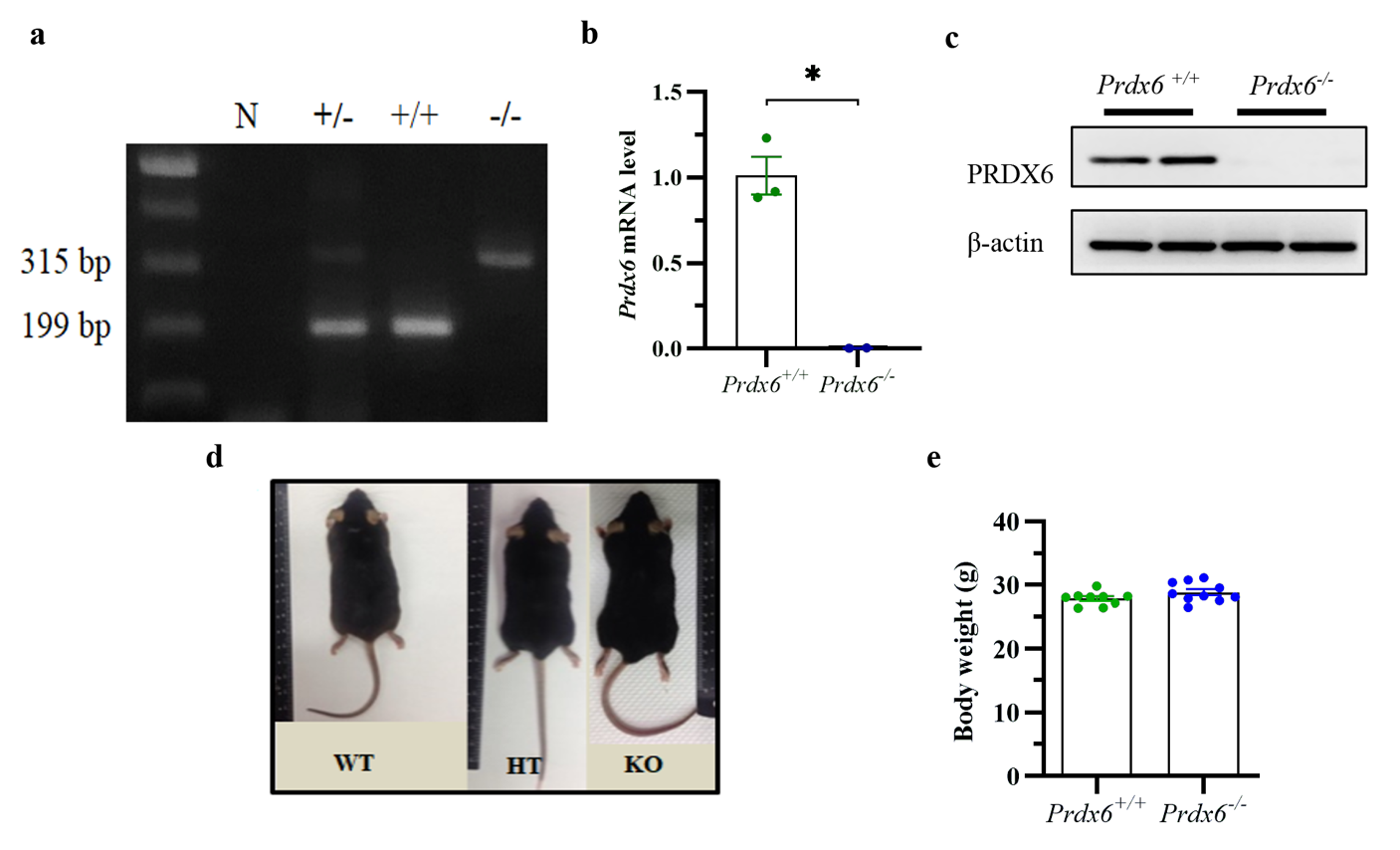
**

**Figure S1.** The characteristics of *Prdx6^-/-^* mice. **(a)** Genotyping of wild-type (WT), heterozygous knockout (HT) and homozygous knockout (KO) mice, respectively. **(b)** Quantification data of mRNA expression of *Prdx6* (*t*_3_ = 7.062, *p* = 0.006). (**c)** Immunoblots showing an absence of PRDX6 in knockout mice. **(d)** Images showing similar morphology between genotypes. **(e)** The body weight of *Prdx6^-/-^* mice is equal to *Prdx6^+/+^* mice (*t*_19_ = -1.426, *p* = 0.170). All data represent the mean ± the SEM. N, negative control; PRDX6, peroxiredoxin 6.

**
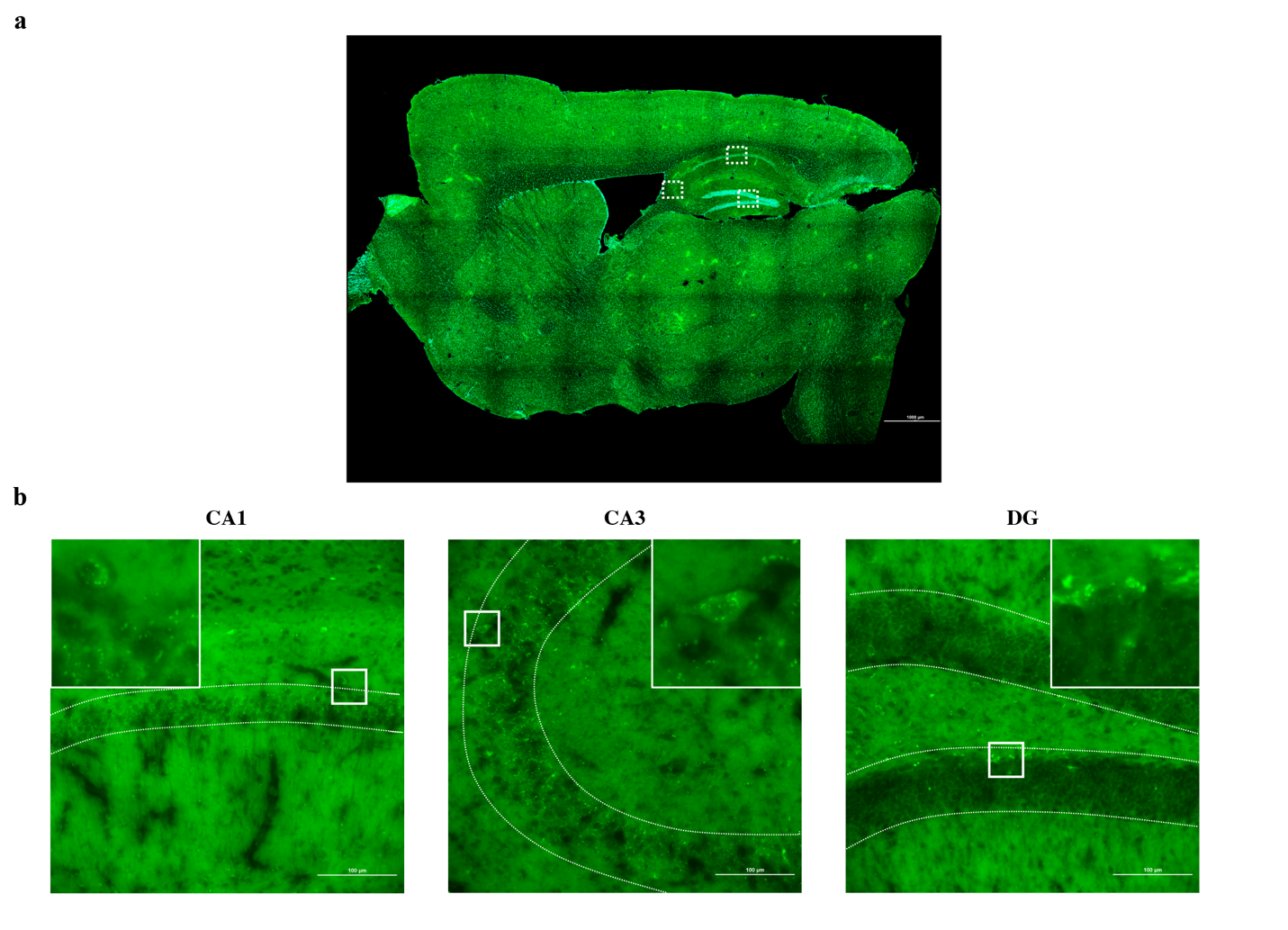
**

**Figure S2.** The expression of EGFP in the hippocampus of *Prdx6^-/-^* mice 4 weeks after intraventricular injection of lentivirus containing EGFP. **(a)** The illustration represents sagittal section of the brain injected with lentivirus containing EGFP. **(b)** Expression of EGFP (green) in CA1, CA3 and DG subregions of the hippocampus.

**
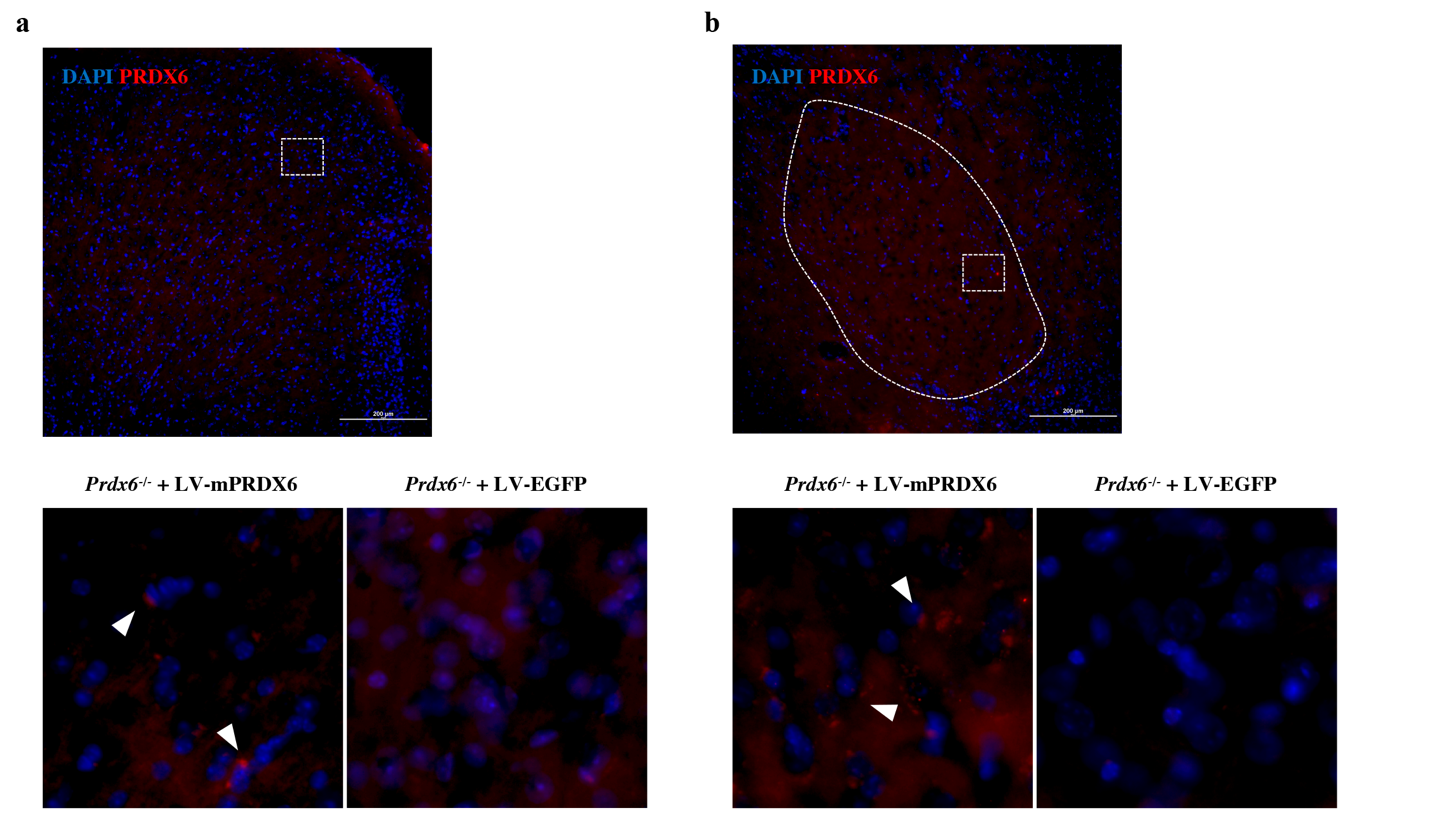
**

**Figure S3.** Distribution of mPRDX6 in the amygdala and prefrontal cortex. **(a and b)** The illustration represents expression level of PRDX6 (red and white arrowhead) in the prefrontal cortex (a) and amygdala (b) of *Prdx6^-/-^* injected with LV-mPRDX6.

**
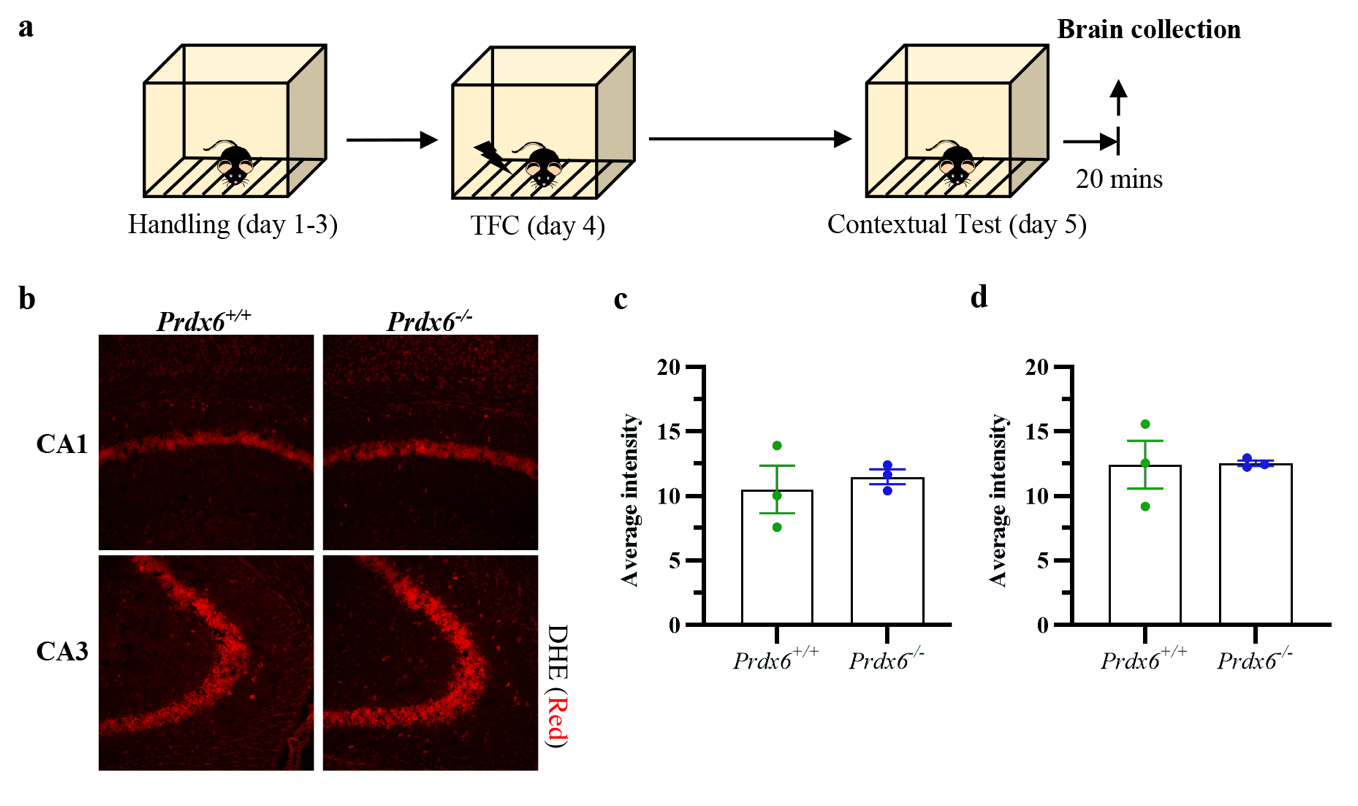
Figure S4.** Superoxide-sensitive DHE staining in hippocampal CA1 and CA3 regions of *Prdx6^+/+^* and *Prdx6^-/-^* mice. **(a)** Brains were isolated 20 minutes after the contextual test. ROS levels in subfields of the hippocampus during contextual fear memory retrieval remain the same. **(b)** Fluorescence images of the hippocampal subregions, including CA1 and CA3, were stained with DHE. **(c and d)** Quantification data showing similar ROS levels in the hippocampal CA1 (n = 3/group) and CA3 of *Prdx6^-/-^* mice in comparison with *Prdx6^+/+^* mice, respectively. All data represent the mean ± the SEM. **p* < 0.05. CA1, cornu ammonis 1; CA3, cornu ammonis 3; DG, dentate gyrus.


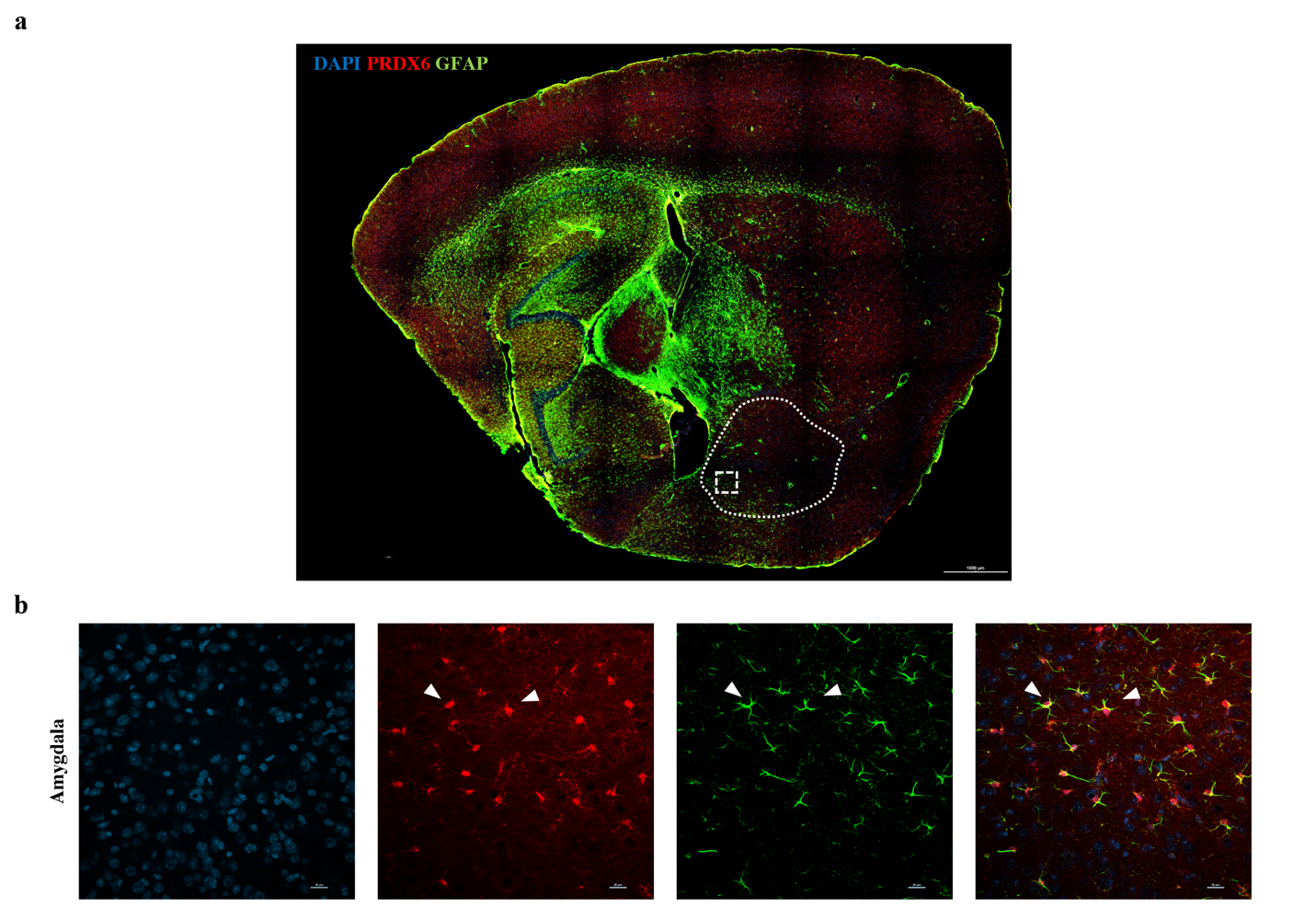


**Figure S5.** High expression level of PRDX6 in the astrocytes of the amygdala. **(a)** The illustration represents sagittal section of the wild-type brain. **(b)** Co-localizations (arrowhead) of PRDX6 (red) with astrocytic marker, GFAP (green) in the amygdala were identified by confocal microscopy.


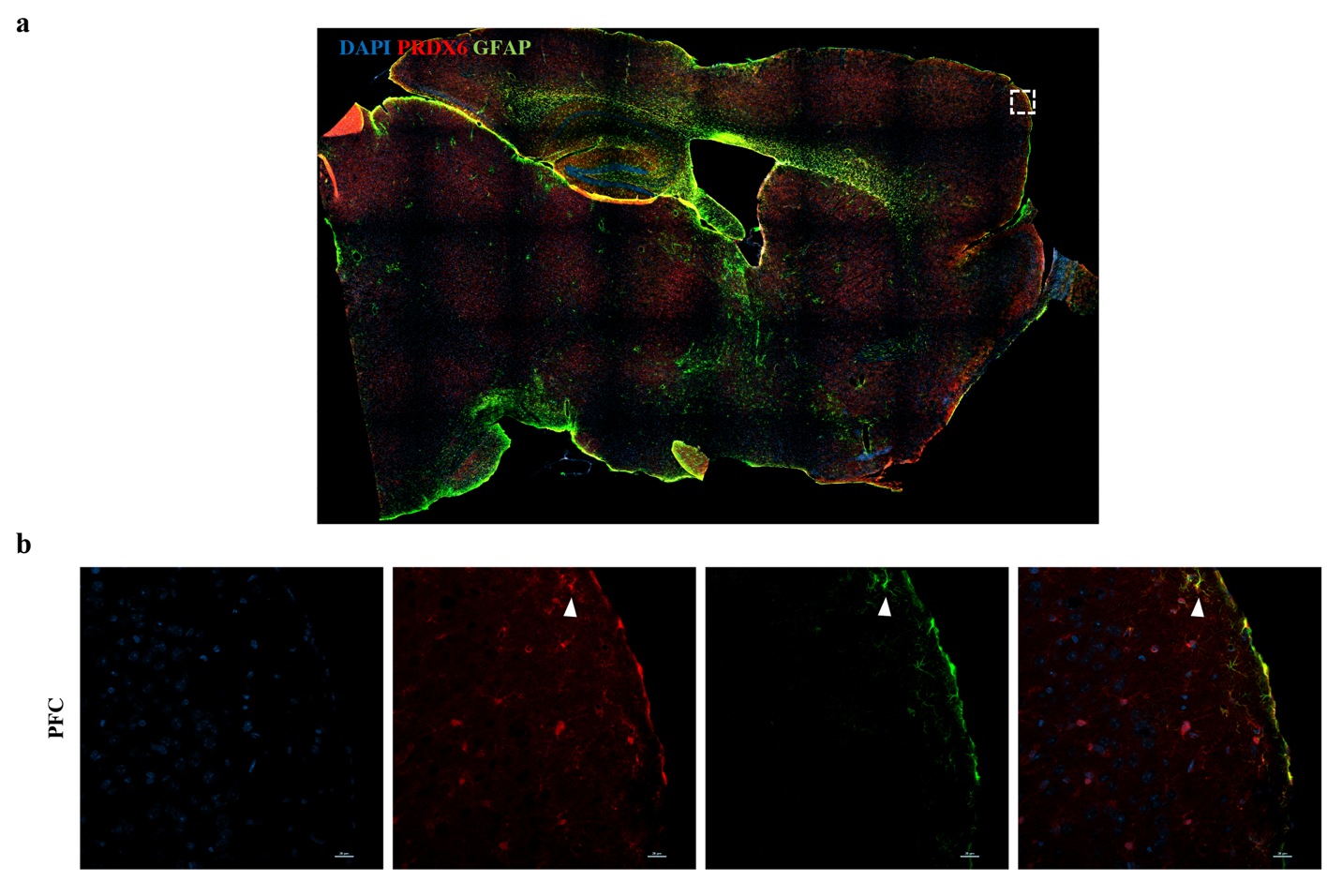


**Figure S6.** Expression level of PRDX6 and GFAP in the prefrontal cortex. **(a)** The illustration represents sagittal section of wild-type brain. **(b)** Expression of PRDX6 (red) and GFAP (green) in the prefrontal cortex. Confocal microscopy was used to determine co-localization of PRDX6 with GFAP (arrowhead).
